# The Neglected Parameter: Tricuspidal E Wave Deceleration Time as Predictor of Atrioventricular Synchrony in VDD Leadless Pacemakers

**DOI:** 10.1111/jce.16613

**Published:** 2025-03-10

**Authors:** Mattia Strazzanti, Caterina Maffeis, Luca Tomasi, Sofia Capocci, Bruna Bolzan, Elena Franchi, Flavio Luciano Ribichini, Giacomo Mugnai

**Affiliations:** ^1^ Cardiology Division, Department of Cardiac, Thoracic and Vascular Sciences Carlo Poma Hospital Mantua Italy; ^2^ Cardiology Division, Department of Medicine, School of Medicine University of Verona Verona Italy

**Keywords:** clinical: implantable devices – pacemaker‐bradyarrhythmias

## Abstract

Leadless pacemakers as the Medtronic Micra AV, have improved cardiac pacing by reducing complications associated with traditional systems. However, achieving high atrioventricular synchrony (AVS) remains a challenge, especially in patients with a high pacing burden. This prospective study enrolled 30 patients to assess the role of echocardiographic parameters in predicting AVS postimplantation. AVS was evaluated via 24‐h Holter monitoring, with a median AVS of 67.9% ± 9.6%. Right atrial function, particularly the tricuspidal E wave deceleration time, emerged as the only independent predictor of AVS (*p* = 0.03), with an AUC of 0.77. These findings suggest that pre‐implantation echocardiographic assessment of right atrial parameters could aid in selecting patients who would benefit most from Micra AV. Further studies with larger cohorts and extended follow‐ups are warranted.

## 1 Introduction

Leadless pacemakers have revolutionized cardiac pacing by eliminating the risks associated with traditional pacemakers [1].

The Micra AV (Medtronic Inc. Minneapolis, MN, US), a second‐generation leadless pacemaker [2], introduces atrioventricular (AV) synchrony through mechanical atrial sensing via an integrated accelerometer [3, 4].

Micra AV is indicated for VDD pacing in patients when a dual chamber transvenous pacing system is considered a poor option (e.g., tortuous anatomy, need to preserve venous access, or increased risk of infection).

However, in clinical practice, the challenge remains in achieving high percentages of AV synchrony [5, 6], especially in patients with an expected high pacing burden, such as those in complete or high‐grade AV block at the baseline ECG.

This study sought to identify the role of the echocardiographic parameters in predicting AV synchrony (AVS) in patients receiving Micra AV pacemakers.

## 2 Methods

This observational study prospectively enrolled 30 patients who received the Micra AV leadless pacemaker at Azienda Ospedaliera Universitaria Integrata, Verona, Italy, between October 2020 and December 2023. Inclusion criteria were patients in sinus rhythm with no requirement for atrial pacing. Exclusion criteria included permanent atrial fibrillation and pregnancy. A comprehensive echocardiographic evaluation was performed before implantation, focusing on both standard parameters and right atrial function. The study was approved by the local medical research ethics committee.

After about 1 month postimplantation, AV synchrony was assessed using a 24‐h Holter electrocardiogram. AVS was defined as a cardiac cycle where the P wave preceded the QRS complex up to 300 ms.

## 3 Results

The mean age of the study population was 74.3 ± 8.5 years (16% female patients). No significant complications occurred during device implantation and all the procedures were successful. The median AVS during predominantly paced episodes was 67.9% ± 9.6%. The study population was then stratified into two groups: Group 1 with AVS > 65% (synchronized) and Group 2 with AVS < 65% (poorly‐synchronized) (Table 1).

**Table 1:** Comparison between the synchronized and nonsynchronized groups based on key clinical and echocardiographic parameters.

Parameter	Synchronized group (*n* = 20)	Nonsynchronized group (*n* = 10)	*p* value
**Age (years)**	73.0 ± 7.6	76.3 ± 10.1	0.34
**Male sex (*n*)**	13 (72%)	10 (100%)	0.23
**BMI**	28.6 ± 8.1	27.0 ± 6.3	0.61
**Chronic kidney failure (*n*)**	4 (22.2%)	5 (50%)	0.21
**Hypertension (*n*)**	14 (77.8%)	7 (70%)	0.67
**Paroxysmal atrial fibrillation (*n*)**	7 (38.9%)	4 (40%)	0.63
**LVEF (%)**	56.9 ± 6.7	55.1 ± 8.3	0.53
**LA volume index (mL/m²)**	43.3 ± 12.4	46.7 ± 11.0	0.49
**RA volume index (cm³/m²)**	29.3 ± 10.1	36.9 ± 6.8	0.04
**TAPSE (mm)**	22.3 ± 3.4	22.0 ± 4.9	0.83
**Mitral E wave (m/sec)**	0.84 ± 0.25	0.90 ± 0.33	0.56
**Mitral A wave (m/sec)**	0.93 ± 0.21	0.92 ± 0.19	0.89
**Mitral E/A ratio**	1.02 ± 0.72	1.00 ± 0.35	0.94
**Mitral E wave deceleration time (ms)**	292.0 ± 50.9	284.4 ± 33.3	0.68
**Tricuspid E wave (m/sec)**	0.78 ± 0.25	0.81 ± 0.37	0.79
**Tricuspid A wave (m/sec)**	0.93 ± 0.33	0.64 ± 0.38	0.04
**Tricuspid E/A ratio**	1.02 ± 0.36	1.06 ± 0.41	0.78
**Tricuspid E wave deceleration time (ms)**	244.9 ± 61.1	182.0 ± 45.5	0.009
**Tricuspid E wave deceleration time (corrected, ms)**	236.8 ± 60.4	174.4 ± 50.5	0.01
**Right atrial strain (%)**	28.6 ± 10.8	33.4 ± 21.4	0.43
**Pulmonary Arterial Pressure (mmHg)**	28.5 ± 8.2	30.0 ± 8.4	0.65

The group with AVS > 65% included 66% of the patients, while 33% had AVS < 65%.

Of note, previous studies found better values of median AVS, but in an artificial setting.

The negative effect of standing and walking on the accelerometer signal quality as well as differences in the heart rate might probably explain the lower values of 24‐h AVS in our real‐world outpatient setting (Table 2).

**Table 2:** Univariate and multivariate analyses of factors predicting AVS in the study.

Parameter	Univariate analysis (OR ± 95% CI)	*p* value	Multivariate analysis (OR ± 95% CI)	*p* value
**Age**	0.95 (0.86–1.05)	0.33	—	—
**BMI**	1.03 (0.92–1.15)	0.59	—	—
**CHA2DS2‐VASc score**	0.70 (0.39–1.23)	0.21	—	—
**HAS‐BLED score**	0.61 (0.21–1.77)	0.36	—	—
**TAVR**	0.73 (0.34–1.87)	0.51	—	—
**Smokers**	1.05 (0.90–1.11)	0.85	—	—
**Chronic kidney failure**	0.35 (0.22–1.44)	0.15	—	—
**Hypertension**	1.07 (0.91–1.27)	0.65	—	—
**Dyslipidemia**	0.82 (0.52–1.94)	0.21	—	—
**Diabetes**	0.31 (0.18–1.49)	0.19	—	—
**LVESV Index (mL/m²)**	0.99 (0.90–1.08)	0.79	—	—
**LVEDV index (mL/m²)**	0.99 (0.95–1.04)	0.75	—	—
**LVEF (%)**	1.04 (0.93–1.16)	0.51	—	—
**LA volume index (mL/m²)**	0.98 (0.91–1.06)	0.49	—	—
**RA volume index (cm³/m²)**	0.91 (0.83–0.99)	0.05	—	—
**Tricuspid A wave (m/sec)**	9.74 (1.02–10.31)	0.05	8.04 (0.62–10.48)	0.11
**Tricuspid E wave deceleration time (ms)**	1.02 (1.01–1.03)	0.02	1.02 (1.00–1.03)	0.03

Some echocardiographic parameters, particularly those related to the right atrial function, showed a significant association with AVS. The right atrial volume index (*p* = 0.04) and tricuspid A wave (*p* = 0.04) were higher in the synchronized group. Notably, the right E wave deceleration time emerged as the only independent predictor of AVS. In detail, the mean tricuspidal E wave deceleration time was 244.9 ms ± 61.1 in the synchronized group and 182.0 ms ± 45.5 in the non‐synchronized group (*p* = 0.009). Correcting this parameter according to the heart rate, using a QTc‐style formula, further supported its significance (*p* = 0.01).

On multivariate analysis, the tricuspidal E wave deceleration time (corrected by the above mentioned formula) was confirmed as the only independent predictor of AVS (*p* = 0.03).

As a variable, the E wave deceleration time corrected for the heart rate was analyzed through a ROC (Receiver Operating Characteristic) curve, setting the outcome as AVS > 65%. The AUC (Area Under the Curve) was 0.77 (95% CI 0.59–0.96, *p* = 0.019), with the best cut‐off value at 193.5 msec (sensibility 77.8%, specificity 70%).

In contrast, traditional factors like right atrium strain or right atrial volume were not found to significantly predict AVS.

## 4 Discussion

Achieving optimal AV synchrony is crucial for improving cardiac performance and minimizing the risk of pacemaker syndrome. Our study identified a few echocardiographic parameters which might be useful to predict which patients are most likely to benefit from Micra AV pacemakers.

The significant relationship between right atrial function and AVS highlights the importance of a pre‐implantation echocardiographic evaluation for selecting the appropriate candidates for leadless pacing. The right atrial E wave deceleration time, in particular, proved to be a good predictor of AV synchrony: this could be explained by the time involved in the perception of the atrial kick by the accelerometer (echocardiographic A wave by mitral pulsed Doppler) of the Micra AV. High filling pressure during the ventricular diastole (resulted in a prolonged E wave deceleration time) could allow the device accelerometer to properly distinguish the two components of the atrial systole.

This suggests that the focus should shift towards the right‐sided heart function when evaluating patients for leadless pacemaker implantation.

This study has several limitations. The small sample size and the single‐center design limit the generalizability of the results. Moreover, the follow‐up period was relatively short, as the AVS was assessed 1 month after the device implantation. Longer follow ups are necessary to determine whether the identified predictors remain consistent over time.

**Figure 1:**
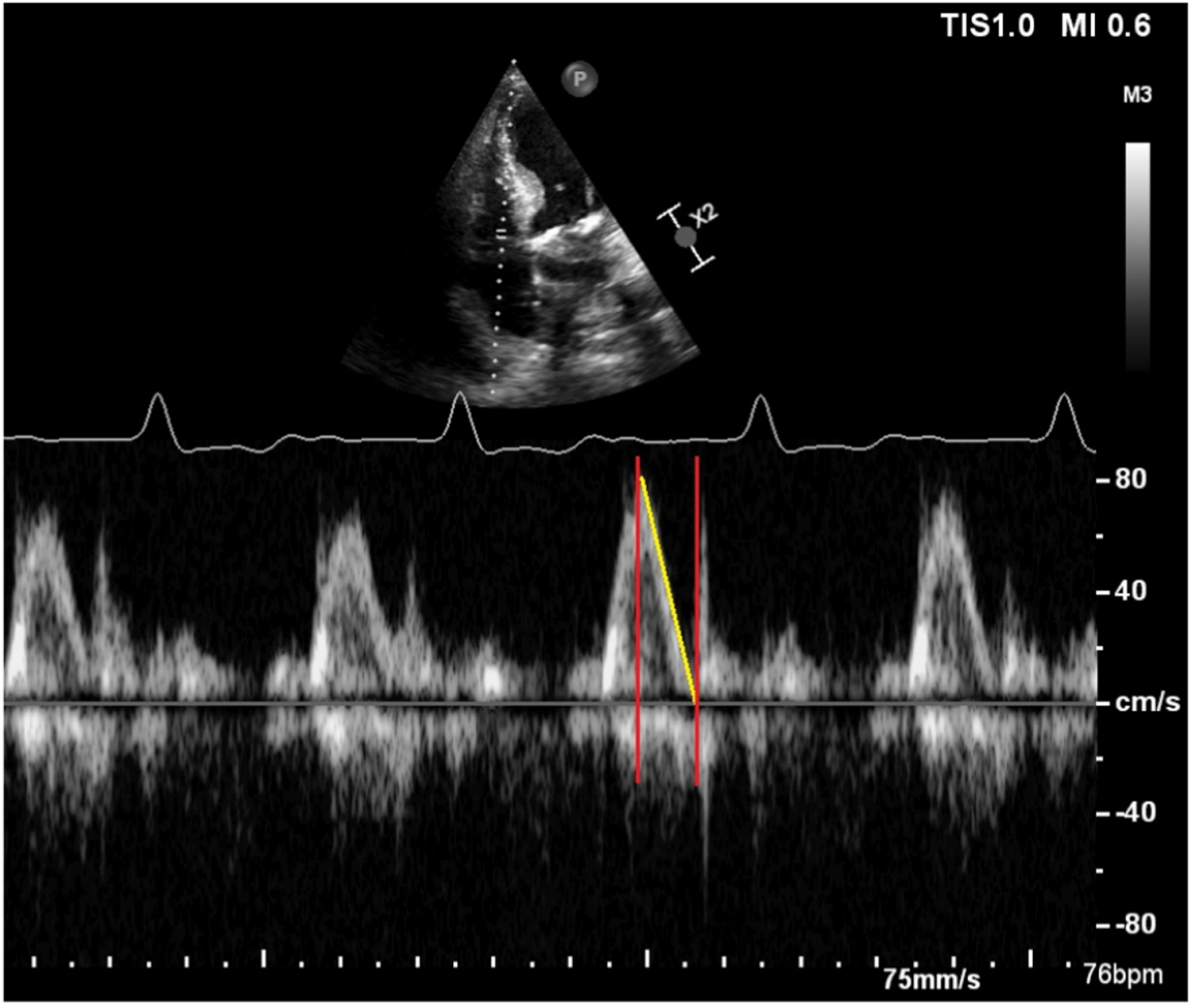
Tricuspidal E wave deceleration time.

In conclusion, this study identified the right atrial function, particularly the right E wave deceleration time, as a significant predictor of AVS in patients receiving Micra AV leadless pacemakers. A pre‐implantation echocardiographic evaluation of the right atrial parameters might help to identify patients who are most likely to benefit from this technology (Figure 1).

Further studies with larger patient cohorts and longer follow ups are needed to validate these findings and refine the patient selection criteria, matching echocardiographic examination with other well‐know parameters of AVS such as as A4‐wave amplitude, ventricular pacing burden, device programming, and sensing vectors.

## Data Availability

The authors have nothing to report.
